# The impact of presence of Hashimoto's thyroiditis on diagnostic accuracy of ultrasound‐guided fine‐needle aspiration biopsy in subcentimeter thyroid nodules: A retrospective study from FUSCC

**DOI:** 10.1002/cam4.997

**Published:** 2017-04-05

**Authors:** Lili Gao, Ben Ma, Li Zhou, Yu Wang, Shuwen Yang, Ning Qu, Yi Gao, Qinghai Ji

**Affiliations:** ^1^ Department of OncologyShanghai Medical CollegeFudan UniversityShanghaiChina; ^2^ Department of PathologyFudan University Shanghai Cancer CenterShanghaiChina; ^3^ Department of Head and Neck SurgeryFudan University Shanghai Cancer CenterShanghaiChina; ^4^ Department of UltrasoundFudan University Shanghai Cancer CenterShanghaiChina

**Keywords:** FNA, Hashimoto's thyroiditis, subcentimeter nodules, thyroid cancer

## Abstract

The incidence of PTMC has been increasing in the recent years. This study aimed to investigate the diagnostic value of US‐FNA in thyroid nodules ≤1 cm and whether the presence of Hashimoto's thyroiditis (HT) in thyroid could influence the accuracy. The patients who accepted US‐FNA at FUSCC from December 2012 to November 2015 and followed our criteria were enrolled in this study. We extracted the cytological, pathological, and follow‐up US/US‐FNA data of patients with subcentimeter nodules. Sensitivity, specificity, positive predictive value (PPV), negative predictive value (NPV), false‐negative rate (FNR), false‐positive rate (FPR), and AUC were calculated to define FNA diagnostic performance in patients. The association of HT with cytological results was analyzed in univariate and multivariate logistic regression analysis. In total, 754 patients with 817 subcentimeter nodules were collected to comprise the FUSCC cohort. Of the 817 nodules, the cytological results were ND/UNS in 80 nodules (9.8%), benign in 74 (9.1%), AUS/FLUS in 80 (9.8%), FN/SFN in 6 (0.7%), suspicious for malignancy (SM) in 222 (27.2%), and malignant in 355 (43.5%). The sensitivity, specificity, PPV, NPV, and AUC of US‐FNA for the subcentimeter nodules were 98.8%, 90.5%, 98.8%, 90.5%, and 94.7%, respectively. In comparison with HT‐positive subcentimeter nodules, the diagnostic value of US‐FNA for HT‐negative nodules was significantly higher (HT‐positive: AUC = 91.6%, HT‐negative: AUC = 95.9%, *P *=* *0.028). The coexistent HT was found to increase the risk of the FNR and indeterminate cytological results. US‐FNA demonstrated an effective method for diagnosis of subcentimeter thyroid nodules with a low nondiagnostic rate in our study. The presence of HT in thyroid could be a risk factor for the increased FNR and indeterminate cytological results during US‐FNA.

## Introduction

Thyroid nodules are a common clinical problem. Due to the widespread use of high‐resolution ultrasound (US), a large number of nonpalpable nodules ≤1 cm have been detected. Fine‐needle aspiration (FNA) biopsy is the most accurate and cost‐effective method for the preoperative diagnosis of thyroid nodules, but the accuracy of FNA performance has varied among different nodules 1, 2, 3, 4. In the American Thyroid Association (ATA) guidelines, FNA is not routinely recommended in patients with nodules ≤1 cm 5. This issue may attribute to the controversial clinical significance for papillary thyroid microcarcinoma (PTMC) on one aspect 6, 7, 8 and the decreased accuracy of FNA in lower size nodules on the other aspect 9. In the guidelines of the American Association of Clinical Endocrinologists (AACE), FNA is recommended for nodules with suspicious malignant US features regardless of the nodule size 10. For physicians, the clinical importance of subcentimeter nodules may predominantly depend on the need to exclude thyroid malignancy due to patient preference and clinical risks.

To optimize patient care and the diagnostic accuracy of US‐FNA, physicians should understand potential factors that influence cytological diagnosis. Moon HI et al. suggested that US‐guided FNA (US‐FNA) performance demonstrated good accuracy in subcentimeter nodules assuming the specimen is adequate, and the decreased nodule size caused the increased rates of inadequate specimens and false‐positive results 9. Patients with subcentimeter nodules sonographically suspicious for malignancy would be recommended to undergo US‐FNA at Fudan University Shanghai Cancer Center (FUSCC) if the patients prefer to exclude thyroid malignancy or have high disease risk factors such as family history of thyroid cancer, radiation history, age more than 45 years and evidence of extrathyroidal extension and lymph node metastasis, etc.

Hashimoto's thyroiditis (HT) is the most common inflammatory thyroid disease, and coexistence of HT with papillary thyroid cancer (PTC) has been widely reported worldwide 11, 12, 13. It remains unclear whether HT background could influence the FNA performance in patients with subcentimeter nodules. This study aimed to retrospectively investigate the impact of presence of HT on the diagnostic accuracy of US‐FNA in subcentimeter nodules.

## Materials and Methods

### Subjects

All the study subjects were patients who accepted US‐FNA at FUSCC from December 2012 to November 2015. The patients included in this study met the following criteria: (1) with thyroid nodule ≤1 cm, (2) undergoing initial US‐FNA performance, (3) no thyroid surgery history before US‐FNA procedures, (4) with test results of serum antithyroperoxidase antibodies (TPOAb) and antithyroglobulin antibodies (TgAb) or postoperative pathological diagnosis of HT, (5) availability of FNA specimen evaluation and adequate medical records.

### US‐FNA performance and cytological diagnosis

US‐FNA was performed by several experienced radiologists. A perpendicular puncture without local anesthesia by a 22‐gauge needle attached to 10 mL plastic syringe was conducted under US guidance. US imaging was presented by using an Acuson Sequoia 8‐15‐MHz linear probe (Siemens Medical Solutions, Mountain View, CA). The aspiration procedure was performed on the suspicious maximum‐size nodule by using the “mixed sampling technique”, that was the operator used his or her wrist to move the needle up and down for a few seconds 14. Three smears were obtained from each nodule and were sent for cytopathological diagnosis. All cytological smears were evaluated by two expert cytopathologists according to the Bethesda System for Reporting Thyroid Cytology 15. Cytopathological diagnosis was divided into six categories: (A) nondiagnostic/unsatisfactory (ND/UNS); (B) benign; (C) atypia/follicular lesion of undetermined significance (AUS/FLUS); (D) follicular neoplasm/suspicious for follicular neoplasm (FN/SFN); (E) suspicious for malignancy (SM); (F) malignant. Indeterminate category was defined as a cytology reading to report AUS/FLUS, FN/SFN or SM 5, 16.

### Clinical data

We reviewed the electronic medical records for collection of clinical, laboratory, radiological, and pathological data. The data on patients' clinical information (gender and age), US features (maximum size of nodule, nodule position, color doppler flow signal, echogenicity, and calcification), laboratory results (serum TPOAb and TgAb levels), cytopathological results, and histological characteristics (histological types, maximum size of foci, and HT) were abstracted from patient records. In our study, HT diagnosis was confirmed by histological pathology in the majority of patients and was made predominantly based on serum TPOAb and TgAb levels for those patients who did not undergo surgery 17.

### Statistical analysis

Categorical data were summarized with frequencies and percentages. The continuous results were expressed as the mean ± standard deviation (SD). Paired‐t and independent‐t test was used to compare continuous variables in two groups. Associations between continuous variables and categorical variables were evaluated using Mann–Whitney U‐tests for two groups and Kruskal–Wallis tests for more than two groups. *χ* ^2^ and Fisher's exact test were used for categorical variables. Sensitivity, specificity, positive predictive value (PPV), negative predictive value (NPV), and AUC (area under the ROC curve; ROC, receiver operating characteristic) were used to define FNA diagnostic performance in patients with benign, SM, and malignant cytology and a corresponding postoperative histological result/a follow‐up US/FNA result. The false‐negative rate (FNR) was calculated as the proportion of histopathologically malignant nodules which obtained benign cytology readings. Moreover, univariate and multivariate analyses were performed to determine whether presence of HT was a risk factor for ND/UNS, indeterminate and false‐negative results in FNA performance, using a logistic regression calculated by odds ratio (OR) and 95% confidence interval (CI). A *P* < 0.05 was considered significant. Statistical analyses were performed using SPSS for Windows (SPSS Inc., Chicago, IL) and STATA version 12.0 (Stata Corp LP, Lakeway Drive, College Station, TX).

## Results

### Basic characteristics of patients and cytological outcomes

**Figure 1:**
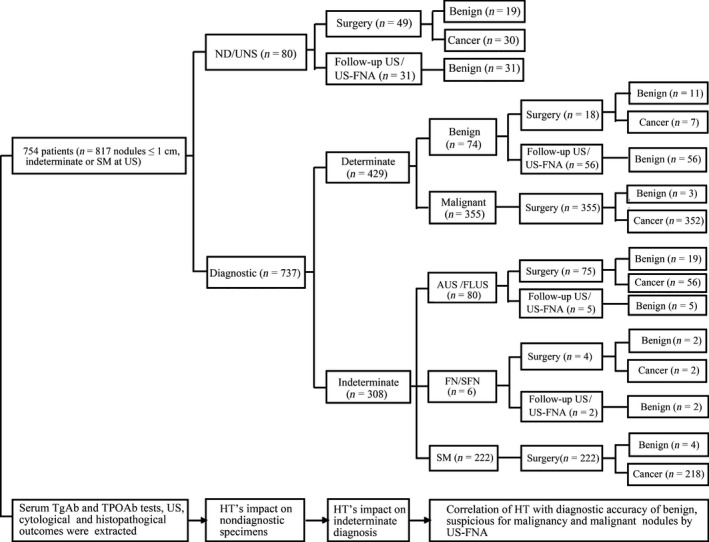
The flow graph of inclusion and analysis of study subjects in our study.

We retrospectively collected and analyzed the records of consecutive patients with subcentimeter nodules indeterminate for diagnosis or suspicious for malignancy at US image who underwent US‐FNA at the FUSCC. The flow graph of inclusion and analysis of study subjects in our study was shown in Figure 1. In total, 754 patients with 817 subcentimeter nodules were collected to comprise the FUSCC cohort. Table 1 summarized the clinical characteristics, US features, cytological results, and histopathological outcomes of the patients enrolled in this study in detail. In 817 nodules, the cytological results were ND/UNS in 80 nodules (9.8%), benign in 74 (9.1%), AUS/FLUS in 80 (9.8%), FN/SFN in 6 (0.7%), SM in 222 (27.2%), and malignant in 355 (43.5%). The 661 patients with 723 nodules accepted surgery and were diagnosed by histological pathology, whereas the other 94 nodules without malignancy evidence at cytology in 93 patients were confirmed by follow‐up US or US‐FNA. Overall, the 665 nodules were found to be malignant, and the other 152 nodules were benign. All of the malignant nodules resected by operators were pathologically diagnosed as PTC.

**Table 1:** Clinicopathological characteristics, US features, and cytological results of patients with subcentimeter nodules in our study

Variables	ND/UNS	Benign	AUS/FLUS	FN/SFN	SM	Malignant	Total
Gender							
Female	64 (9.7%)	62 (9.4%)	69 (10.4%)	5 (0.8%)	175 (26.4%)	287 (43.3%)	662
Male	16 (10.3%)	12 (7.7%)	11 (7.1%)	1 (0.65)	47 (30.3%)	68 (43.9%)	155
Age (years)	48.71 ± 12.34	49.92 ± 9.85	46.65 ± 11.85	53.33 ± 15.85	46.62 ± 11.30	44.51 ± 11.29	46.26 ± 11.49
US features							
Nodule size (cm)							
<0.5	11 (7.3%)	3 (2.0%)	16 (10.6%)	1 (0.7%)	55 (36.4%)	65 (43.0%)	151
≥0.5	69 (10.4%)	71 (10.7%)	64 (9.6%)	5 (0.8%)	167 (25.1%)	290 (43.5%)	666
Echogenicity							
Hypoechogenicity	78 (9.8%)	65 (8.2%)	79 (9.9%)	6 (0.8%)	215 (27.1%)	351 (44.2%)	794
Isoechogenicity	2 (8.7%)	9 (39.1%)	1 (4.3%)	0 (0.0%)	7 (30.4%)	4 (17.4%)	23
Calcification							
None	34 (9.4%)	37 (10.2%)	33 (9.1%)	4 (1.1%)	94 (26.0%)	160 (44.2%)	362
Microcalcification	32 (9.4%)	27 (7.9%)	30 (8.8%)	2 (0.6%)	94 (27.6%)	156 (45.7%)	341
Macrocalcification	14 (12.3)	10 (8.8%)	17 (14.9%)	0 (0.0%)	34 (29.8%)	39 (34.2%)	114
Intranodular vascularity							
No	70 (10.3%)	61 (9.0%)	64 (9.5%)	4 (0.6%)	182 (26.9%)	296 (43.7%)	677
Yes	10 (7.1%)	13 (9.3%)	16 (11.4%)	2 (1.4%)	40 (28.6%)	59 (42.1%)	140
HT							
Negative	63 (10.7%)	50 (8.5%)	52 (8.8%)	4 (0.7%)	149 (25.3%)	272 (46.1%)	590
Positive	17 (7.5%)	24 (10.6%)	28 (12.3%)	2 (0.9%)	73 (32.2%)	83 (36.6%)	227
Surgery							
Yes	49 (6.8%)	18 (2.5%)	75 (10.4%)	4 (0.6%)	222 (30.7%)	355 (43.5%)	723
No	31 (33.0%)	56 (59.6%)	5 (5.3%)	2 (2.1%)	0 (0.0%)	0 (0.0%)	94
Pathology or follow‐up US/US‐FNA							
Benign	50 (32.9%)	67 (44.1%)	24 (15.8%)	4 (2.6%)	4 (2.6%)	3 (0.8%)	152
Malignant	30 (4.5%)	7 (1.1%)	56 (8.4%)	2 (0.3%)	218 (32.8%)	352 (52.9%)	665
Total	80 (9.8%)	74 (9.1%)	80 (9.8%)	6 (0.7%)	222 (27.2%)	355 (43.5%)	817

US, ultrasound; ND/UNS, nondiagnostic/unsatisfactory; AUS/FLUS, atypia/follicular lesion of undetermined significance; FN/SFN, follicular neoplasm/suspicious for follicular neoplasm; SM, suspicious for malignancy; HT, Hashimoto's disease; US‐FNA, ultrasound‐guided fine‐needle aspiration.

### Association between HT and cytological and pathological results

**Figure 2:**
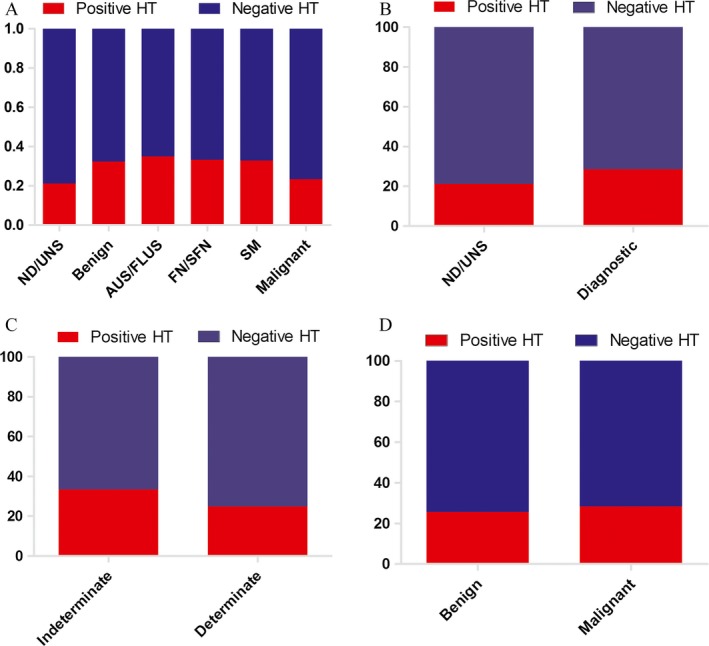
Correlations of presence of Hashimoto's thyroiditis (HT) with cytological and pathological results. (A) The positive rates of HT in ND/UNS, benign, AUS/FLUS, FN/SFN, SM, and malignant groups were, respectively, 21.2% (17/80), 32.4% (24/74), 35.0% (28/80), 33.3% (2/6), 32.9% (73/222), and 23.4% (83/355), (B) The HT‐positive rate between the ND/UNS group (21.2%) and the diagnostic group (28.5%) showed no significant difference (*P *=* *0.170). (C) The concurrence rate of HT in indeterminate nodules (103/308, 33.4%) was significantly higher than in determinate nodules (107/429, 24.9%, *P *=* *0.012). (D) The coexistent rate of HT showed no significant difference (*P *=* *0.516) between benign (25.7%) and malignant (28.3%) nodules confirmed by pathology.

HT was coexistent with 227 nodules and accounted for 27.8% in all (Table 1). Figure 2A showed that HT‐positive rates of ND/UNS, benign, AUS/FLUS, FN/SFN, SM, and malignant groups were, respectively, 21.2% (17/80), 32.4% (24/74), 35.0% (28/80), 33.3% (2/6), 32.9% (73/222), and 23.4% (83/355). According to the evaluation of sample adequacy and satisfaction for diagnosis, the cytological specimens were grouped into the ND/UNS group and the diagnostic group (benign, AUS/FLUS, FN/SFN, SM, and malignant). Our findings suggested there was no significant difference in HT‐positive rate between the ND/UNS group (21.2%) and the diagnostic group (28.5%, *P *=* *0.170, Fig. 2B). Then, we performed a further comparative analysis in frequency of presence of HT among the five cytological categories in the diagnostic group. Due to the existence of indeterminate cytology, we categorized benign, AUS/FLUS, FN/SFN, SM, and malignant nodules into two types: indeterminate (AUS/FLUS, FN/SFN and SM) and determinate (benign and malignant) categories. The concurrence rate of HT in indeterminate nodules (103/308, 33.4%) was significantly higher than in determinate nodules (107/429, 24.9%, *P *=* *0.012, Fig. 2C). As shown in Figure 2D, the coexistent rate of HT showed no significant difference between benign and malignant nodules confirmed by histopathology.

### Impact of presence of HT on diagnostic values of US‐FNA

We performed an analysis for the diagnostic accuracy of US‐FNA in patients with benign, SM, and malignant cytology and corresponding histological/follow‐up results and further analyzed the impact of HT on the diagnostic value. Table 2 showed that sensitivity, specificity, PPV, NPV, and AUC of US‐FNA for 651 subcentimeter nodules were 98.8%, 90.5%, 98.8%, 90.5%, and 94.7%, respectively. In comparison with HT‐positive subcentimeter nodules, the diagnostic value of US‐FNA for HT‐negative nodules was significantly higher (HT‐positive: AUC = 91.6%, HT‐negative: AUC = 95.9%, *P *=* *0.028). The FPR and FNR of US‐FNA reached 9.5% and 1.2%, respectively and risk factors for false‐negative results were evaluated by univariate regression analysis. As shown in Table 3, presence of HT was the only one risk factor for the increased FNR (*P *=* *0.022, OR=6.83, 95%CI: 1.311–35.573).

**Table 2:** A comparison between HT‐positive subcentimeter nodules and HT‐negative nodules in diagnostic value of US‐FNA

HT	Sensitivity (%)	Specificity (%)	PPV (%)	NPV (%)	FPR (%)	FNR (%)	AUC (95%CI)	*P* value
Positive (*n* = 180)	96.8%	86.4%	98.1%	79.1%	13.6%	3.2%	0.916 (0.83–1.00)	***0.028***
Negative (*n* = 471)	99.5%	92.3%	99.0%	96.0%	7.7%	0.5%	0.959 (0.92–1.00)	
Total (*n* = 651)	98.8%	90.5%	98.8%	90.5%	9.5%	1.2%	0.947 (0.91–0.99)	

Italic and bold type indicates statistical significance.

HT, Hashimoto's thyroiditis; US‐FNA, ultrasound‐guided fine‐needle aspiration; PPV, positive predictive value; NPV, negative predictive value; FPR, false‐positive rate; FNR, false‐negative rate; AUC, area under ROC curve; ROC, receiver operating curve; CI, confidence interval. [Correction added on 19 April 2017, after first online publication: There are several changes in Table 2. The value in FPR (%) and FNR (%) column were previously wrong and these have now been corrected in this version.]

**Table 3:** Univariate analysis of risk factors for false‐negative results of FNA in subcentimeter nodules

Variables	Univariate analysis		
	*P* value	OR	95.0% CI for OR
Female	0.710	1.497	0.178–12.556
Age ≥45	0.407	1.894	0.419–8.554
US features			
Nodule size < 0.5 cm	0.629	1.503	0.288–7.845
Isoechogenicity	0.057	8.485	0.941–76.537
Macrocalcification	0.895	1.155	0.137–9.733
Intranodular vascularity	0.102	3.534	0.778–16.029
HT	***0.022***	6.830	1.311–35.573

Italic and bold type indicates statistical significance.

FNA, fine‐needle aspiration; OR, odds ratio; CI, confidence interval; US, ultrasound; HT, Hashimoto's thyroiditis.

### Risk factors associated with indeterminate diagnosis

The indeterminate rate of FNA reached 37.7% (308/817) in our study. Clinicopathological factors were assessed to identify possible risk factors for increased indeterminate diagnosis as shown in Table 4. The results indicated that nodule size less than 0.5 cm, macrocalcification and presence of HT were significantly correlated with indeterminate cytology. The univariate and multivariate logistic regression analyses in Table 5 indicated the above three factors were confirmed to be independent risk factors for indeterminate cytological results.

**Table 4:** Clinicopathological factors associated with indeterminate results of US‐FNA in subcentimeter nodules

Variables	*N*	Indeterminate		*P* value
		Negative	Positive	
Gender				0.860
Female	598	349 (58.4%)	249 (41.6%)	
Male	139	80 (57.6%)	59 (42.4%)	
Age (years)	737	45.45 ± 11.23	46.76 ± 11.53	0.120
US features				
Nodule size (cm)				***0.010***
<0.5	140	68 (48.6%)	72 (51.4%)	
≥0.5	597	361 (60.5%)	236 (39.5%)	
Echogenicity				0.730
Hypoechogenicity	716	416 (58.1%)	300 (41.9%)	
Isoechogenicity	21	13 (61.9%)	8 (38.1%)	
Calcification				***0.045***
None/Microcalcification	637	380 (59.7%)	257 (40.3%)	
Macrocalcification	100	49 (49.0%)	51 (51.0%)	
Intranodular vascularity				0.472
No	607	357 (58.8%)	250 (41.2%)	
Yes	130	72 (55.4%)	58 (44.6%)	
HT				***0.012***
Absent	527	322 (61.1%)	205 (38.9%)	
Present	210	107 (51.0%)	103 (49.0%)	

Italic and bold type indicates statistical significance.

US‐FNA, ultrasound‐guided fine‐needle aspiration; OR, odds ratio; CI, confidence interval; US, ultrasound; HT, Hashimoto's thyroiditis.

**Table 5:** Logistic regression analysis for risk factors of indeterminate results of US‐FNA

Variables	Univariate analysis			Multivariate analysis		
	*P* value	OR	95.0% CI for OR	*P* value	OR	95.0% CI for OR
Female	0.862	0.967	0.666–1.406			
Age	0.123	1.010	0.997–1.023			
US features						
Nodule size < 0.5 cm	***0.011***	1.620	1.119–2.344	***0.005***	1.717	1.179–2.500
Isoechogenicity	0.728	0.853	0.349–2.085			
Macrocalcification	***0.046***	1.539	1.008–2.349	***0.011***	1.749	1.136–2.692
Intranodular vascularity	0.472	1.150	0.785–1.685			
HT	***0.012***	1.512	1.095–2.087	***0.007***	1.573	1.134–2.180

Italic and bold type indicates statistical significance.

US‐FNA, ultrasound‐guided fine‐needle aspiration; OR, odds ratio; CI, confidence interval; US, ultrasound; HT, Hashimoto's thyroiditis.

## Discussion

According to the 2015 ATA guideline, the yearly incidence of thyroid cancer in the United States has nearly tripled from 4.9 per 100,000 in 1975 to 14.3 per 100,000 in 2009, and almost the entire change has been attributed to an increase in the incidence of PTC 5. Due to the wide use of high‐resolution US and US‐FNA, increasing numbers of patients have been diagnosed as PTMCs. In management of thyroid nodules ≤1 cm, the performance of US‐FNA is widely debated. On one aspect, the clinical implication of PTMCs is still controversial, and on the other aspect the diagnostic accuracy of US‐FNA strongly depends on the intrinsic characteristics of nodules, the experience of operators, cytological preparation, and interpretation of cytopathologists. Although recent studies have suggested that excellent outcomes for most PTMC patients are more related to the indolent nature of the disease rather than to the effectiveness of treatment 8, 18, 19, we have no specific indicators that reliably differentiate the relatively small number of PTMC patients destined to develop clinically significant progression from the larger sample size of patients that will not cause significant disease 5. It is necessary for clinical physicians to exclude malignancy of thyroid nodule due to patients' preference and clinical risks. Therefore, radiologists should understand various factors influencing cytological accuracy of US‐FNA in patients with subcentimeter nodules.

Although some studies have demonstrated that US‐FNA is a useful tool for subcentimeter nodules with good diagnostic accuracy 9, 20, 21, the rates of nondiagnostic FNA results were highly variable and ranged between 0.4% and 17.7% 9, 20, 21, 22, 23, 24. ND means that the slide prepared by US‐FNA contains inadequate or unsatisfactory contents that can be interpreted by cytopathologists. The nondiagnostic rate of US‐FNA in our study was less than 10%, which reached the recommended standard value (less than 10–15%) by the Korean Society of Thyroid Radiology (KSThR) 25. The low nondiagnostic rate should attribute to standardization of US‐FNA technique by experienced radiologists and efficient cytological evaluation based on the Bethesda System for Reporting Thyroid Cytology by cytopathogists. Moreover, cellular specimen processing by conventional smear techniques combined with liquid‐based cytology (LBC) in our study showed the advantage of improving diagnostic yield.

As a common type of inflammatory thyroid disease, HT accounts for 20–25% of thyroid disease patients in China with approximately 0.4–1.5% of the population affected 26, 27. A close relationship between HT and PTC has been reported in large numbers of studies 11, 12, 13, 28, 29, 30, 31. However, the impact of coexistent HT on the diagnostic performance of US‐FNA in subcentimeter nodules remains unclear. For the first time, this study suggested that subcentimeter nodules coexistent with and without HT showed a significant difference in diagnostic accuracy of FNA in the area with a high prevalence of HT. The presence of HT in thyroid significantly decreased sensitivity, specificity, NPV and PPV, and increased FNR of US‐FNA in subcentimeter nodules. Moreover, the indeterminate rate of cytological results was markedly improved due to the coexistence of HT.

The sonographic appearance of HT exhibits diversity and complexity, especially when coexistent with nodules or other disorders. Pedersen OM et al. 32 indicate that a diffuse reduction in thyroid echogenicity is a valid characteristic of HT under US, which may attribute to the pathological basis of inflammatory cell infiltration into thyroid. Wu GH et al. 33 investigate ultrasonographic characteristics of HT based on its pathological changes. In addition to diffuse hypoechogenicity, pseudonodules, and inhomogeneous parenchyma have been observed in patients with HT. Wu GH et al. suggest that fibroplastic proliferation may contribute to the sonographic changes in pseudonodules and inhomogeneous parenchyma when HT occurs in thyroid 33, which probably exert poor impact on accurate identification and aspiration of subcentimeter nodules when US‐FNA is performed and cause the increase in the FNR. Furthermore, morphologic and pathophysiological changes in HT including inflammatory infiltration of the parenchyma, angiectasis and vascular proliferation are possible to increase hemorrhage during the FNA process and to add mixed components into cytological smears, presumably contributing to indeterminate cytological results.

The FPR of the diagnostic FNAs in our series was 9.5% (7/74). The analyses of histopathological results in the seven cases suggested that papillary atypical hyperplasia were found in four cases and adenomas coexistent with inflammatory cells appeared in three cases, which were responsible for the relatively high FNR On reviewing the false‐negative cases we found that one case had a microscopic PTC with a diameter around 1 mm. This was an incidental finding, because the case was operated on for PTC based on the cytologic diagnosis of the other lobe of the thyroid. The cause of false‐negative result in another case was sampling error. The cause of the diagnostic error in the remaining five cases (71.4%) was the coexistence of HT. Kollur SM et al. 34 noted that aspirating on and around the thyroid nodule coexisting with HT helps in sampling HT and may lead to false‐negative results. The retrospective review of the smears in these five cases showed the presence of moderate or excessive numbers of lymphocytes, only a few Hürthle cells and relative paucity of tumor cells. According to the Bethesda System for Reporting Thyroid Cytology 15, the components of FNA in nodules formed by HT may contain only numerous inflammatory cells. Such cases are usually interpreted as benign lesions since a minimum number of follicular cells observed on the smear are not required.

In conclusion, US‐FNA demonstrated an effective method for diagnosis of subcentimeter nodules with a low nondiagnostic rate in our study when experienced operators and standard cytological preparation and evaluation were assumed. The appearance of HT was found to decrease the accuracy and raise the FNR of US‐FNA in subcentimeter nodules, and to significantly cause the increase in indeterminate cytological results.

### Ethics Statement

Each patient provided a written informed consent for his/her specimens and information to be used for research and stored in the hospital database, and this study was approved by the Ethical Committee of the FUSCC. All procedures performed in our study were in accordance with the ethical standards of our institutional research committee and with the 1964 Helsinki declaration and its later amendments or comparable ethical standards.

## Conflict Interest

We declare that there is no conflict of interest that could.
